# Effect of Rice Protein Meal Replacement of Fish Meal on Growth, Anti-Oxidation Capacity, and Non-Specific Immunity for Juvenile Shrimp *Litopenaeus vannamei*

**DOI:** 10.3390/ani12243579

**Published:** 2022-12-17

**Authors:** Huaxing Lin, Shuqing He, Beiping Tan, Xiaomin Zhang, Yi Lin, Qihui Yang

**Affiliations:** 1 College of Fisheries, Guangdong Ocean University, Zhanjiang 524088, China; 2 Aquatic Animals Precision Nutrition and High Efficiency Feed Engineering Research Center of Guangdong Province, Zhanjiang 524088, China; 3 Guangdong Provincial Key Laboratory of Aquatic Animal Disease Control and Healthy Culture, Zhanjiang 524088, China

**Keywords:** *Litopenaeus vannamei*, rice protein meal, fish meal, anti-oxidation capacity, non-specific immunity

## Abstract

**Simple Summary:**

Fishmeal is the most important source of protein in aquafeeds. In recent years, declining fishery resources and increasing demand have led to a shortage of fishmeal resources. It is crucial to find a low-cost, high-quality protein source to replace fishmeal in order to ensure the sustainable development of aquaculture. Rice protein meal is a high-quality plant protein with high protein and fat content and is relatively balanced amino acid content. In the present study, an experiment was carried out to replace fishmeal with rice protein meal in the feed of *Litopenaeus vannamei*. The results showed that it was possible to replace fishmeal with 10% rice protein in a feed containing 20% fishmeal without adversely affecting the growth of *Litopenaeus vannamei* and to some extent improving the immunity of the organism. In addition, the replacement of 10% fishmeal with rice protein meal significantly improves the promotion of digestibility, protein synthesis, antioxidant capacity, and disease resistance of *Litopenaeus vannamei*.

**Abstract:**

This study assessed the effect of rice protein meal replacement for fish meal on the growth, nonspecific immunity, and disease resistance on juvenile shrimp *Litopenaeus vannamei*. Six groups of iso-nitrogenous and iso-lipid feeds named FM, R10, R20, R40, R60, and R80 were prepared by replacing 0%, 10%, 20%, 40%, 60%, and 80% in FM protein with RPM, respectively, and then fed to the shrimps (0.54 ± 0.01 g). An amount of 720 healthy and evenly sized shrimps were allocated to six groups (three replicates per group) and fed four times a day (7:00, 11:00, 17:00 and 21:00) for eight weeks. Results revealed no significant differences in WG, FCR, and SGR of shrimps after replacing FM with 10% RPM (*p* > 0.05). In the R10 and R20 groups, SOD and T-AOC activities were significantly higher than those in the FM group, whereas the opposite was observed for MDA content (*p* < 0.05). CAT, ACP, and LZM were all significantly higher in the R10, R20, and R40 groups than in the FM group (*p* < 0.05). GSH-Px activity in the R10 group was significantly higher than the activity in the FM group (*p* < 0.05). AKP, PO, TYS, GPT, and GOT activities were significantly higher in the R10 group than in the FM group (*p* < 0.05). Compared to the FM group, the eukaryotic translation initiation factor 3K (*eif3k*) gene was significantly up-regulated in the R10 group, whereas the penaiedin 3a (*pen 3a*) and anti-lipopolysaccharide factor (*alf*) genes were significantly up-regulated in the R10 and R20 groups (*p* < 0.05). The crustin a (*cru a*), immune deficiency (*imd*), and lysozyme (*lzm*) mRNA levels were significantly higher in the R10, R20, and R40 groups than in the other groups (*p* < 0.05). The prophenoloxidase (*PO*) mRNA levels in the R20 group were significantly higher than those in the FM group (*p* < 0.05). The replacement of 10–40% of FM with RPM improved the gut flora composition of shrimps, increasing beneficial bacteria (Bacteroidetes) abundance and reducing harmful bacteria (*Aspergillus* and *Vibrio*) abundance. After the challenge test of *Vibrio parahaemolyticus* (7 days), the cumulative mortality in the R10 group significantly decreased (*p* < 0.05). In conclusion, replacement of 10% FM by RPM significantly improved digestibility, protein synthesis, antioxidant capacity, and disease resistance in *L. vannamei*.

## 1. Introduction

Shrimp *Litopenaeus vannamei* production accounts for around 70% of China’s shrimp farming production [1,2]. This species is popular with farmers because of its fast growth rate, adaptability to changes in water environment, and resistance to high-density culture and disease [3]. With rapidly developing intensive aquaculture, the quantity, variety, and production of aquafeeds are rapidly increasing, and the demand for fish meal (FM) has also increased significantly [4,5]. However, global FM production is unable to meet the growing demand for aquaculture, and the continued rise in FM prices and the imbalance between supply and demand in the market have significant negative effects on the development of the aquaculture industry [6]. Therefore, the search for FM alternatives has become a research hotspot for the global aquaculture industry [7].

Rice protein meal (RPM), a high-quality edible protein with high bioavailability, is a low antigenic protein that does not cause allergic reactions [8]. RPM is extracted by separation from processing byproducts such as broken rice, rice germ, rice bran, and rice husk [9]. Its crude protein content is generally 60% to 68%, and its digestible protein content is 56% or more, making it more bio-effective and easier to absorb and utilize than maize, wheat, and other proteins [10]. In addition, rice protein hydrolysates contain a variety of physiologically active small molecule peptides with antioxidant and immunomodulatory activities [11]. RPM is widely sourced, productive, and rich in nutrients [12].

Some studies replaced FM with RPM for *L. vannamei* feed by evaluating the utilization and nutrient digestibility of RPM by shrimp. They found that FM could be replaced with RPM in shrimp feed [13,14]. High levels of vegetable protein are harmful to shrimp health and affect the composition of shrimp gut bacteria [15,16]. The normal flora of the gut is important for the digestive function and health of shrimp. When the intestinal flora enters dysbiosis, the immune system of the host may be affected. Under normal conditions, the dominant flora colonizing the gut acts as a stable ecological community that repels foreign bacteria and serves as an important biological barrier for the immunity of the shrimp gut. However, the effect of RPM on the intestinal flora and immunity of shrimp has not been studied. Therefore, an evaluation of the effects of RPM on growth, non-specific immunity, and intestinal flora for *L. vannamei* was carried out.

## 2. Materials and Methods

### 2.1. Diet Formulation and Preparation

The proportion of FM in the base diet for the present study is 20% (Table 1). Six diets (iso-nitrogenous and iso-lipid) were prepared to contain 0%, 1.80%, 3.61%, 7.21%, 10.82%, and 14.42% of RPM (crude protein content: 66.00%; crude lipid content: 5.65%) by replacing 0% (FM), 10% (R10), 20% (R20), 40% (R40), 60% (R60), and 80% (R80) of FM protein, respectively. The proportion of FM in the feed was reduced by 0%, 2%, 4%, 8%, 12%, and 16%, respectively. The amino acid composition of the diet is displayed in Table 2.

All the materials were crushed, sieved (80-mesh), and mixed. Then, oil and water were added, granulated (1.5 mm diameter), dried, and stored in the fridge (−20 °C) [17]. The machines used were as follows: a hammer mill (SF-320, Suzhong Pharmaceutical Machinery Co., Ltd., Suzhong, Jiangsu Province, China), a V-type vertical mixer (JS-14S; Zhejiang Zhengtai Electric Co., Ltd., Zhejiang Province, China), and a double screw extruder (F-75; South China University of Technology, Guangdong Province, China) [18].

### 2.2. Animals and Management

The trials were carried out at an experimental site in the High Technology Park of Guangdong Ocean University, China. Juvenile *L*. *vannamei* came from the South Marine Aquaculture Seed Base of Zhanjiang Hengxing South Marine Technology Ltd. Co., China. Live *L*. *vannamei* were transported using transport techniques and cultured in 0.3 m^3^ glass fiber barrels [19]. In the early stage of the experiment, the shrimps were fed a commercially available diet (48% crude protein, 8% crude lipid) for 14 d. In the present experiment, 720 shrimps (0.54 ± 0.01 g) were randomly assigned to six groups and separated into glass fiber buckets (0.3 m^3^). Each group was fed the corresponding feed four times a day (7:00, 11:00, 17:00 and 21:00), stopping at a significant satiety level. The shrimps were observed before each feeding and then fed for eight weeks. After 40 min, the shrimps were observed eating and the amount of feed was adjusted. The amount of feed consumed, the water temperature (28–31 °C), and the salinity (25–30 ppt) were recorded daily.

### 2.3. Sample Collection

The shrimp samples maintained fasting for 24 h before collection and anaesthetization with MS-222 (1:10,000) [16]. Each group of shrimps was counted and weighed for analysis of growth performance. Afterwards, three shrimp samples were selected for whole body composition. Blood samples were collected and then kept at 4 °C. After centrifugation (3500 rpm, 4 °C, 15 min), the serum was obtainable and immediately stored in a refrigerator (−80 °C) for analysis [20].

At the end, the hepatopancreas of three shrimp was randomly taken after 24 h of starvation. The samples were weighed, 9 times volume of frozen buffer at 4 °C was added, homogenized, left overnight in a fridge (4 °C), and centrifuged at 3000 rpm/min for 15 min. The supernatant was collected and stored in a fridge (4 °C) in preparation for testing the relevant enzyme activity. Three shrimps were selected, rapidly removed from the hepatopancreas, placed in an RNA-later and stored a refrigerator (−80 °C) for the determination of relevant gene expression. The intestines of three shrimps were acquired and kept in a refrigerator (−80 °C) for gut microbiota analysis. The samples collected were all randomly selected from each replicate.

### 2.4. Challenge Test

*Vibrio parahaemolyticus* was obtained from Guangdong Provincial Key Laboratory of Pathogenic Biology and Epidemiology for Aquatic Economic Animals (Zhanjiang, China) and was activated twice following the Xie et al. [21] method. At a later stage of the experiment, 15 shrimps were picked at random from each replicate to test for disease resistance. After pre-testing, a semi-lethal concentration (LD_50_, 7 d) of 2.88 × 10^8^ CFU/mL was obtained for *V*. *parahaemolyticus*. An amount of 50 μL of *V*. *parahaemolyticus* was then injected into the dorsal surface of the second to third abdominal segment of the shrimp and the shrimp were observed. The number of shrimp dead at 7 d after bacterial injection was counted and the accumulated mortality rate (%) was calculated.

### 2.5. Formula for Calculations

Weight gain (WG, %) = 100 × (final body weight (g) − initial body weight (g))/initial body weight (g);

Feed coefficient rate (FCR) = feed intake (g)/(final body weight (g) − initial body weight (g));

Specific growth rate (SGR, %/d) = 100 × [ln (final body weight (g)) − ln (initial body weight (g))]/days;

Survival rate (SR, %) = 100 × (final shrimp number/initial shrimp number);

Protein efficiency rate (PER) = weight gain/protein intake;

Protein deposition rate (PDR) = 100 × [final body weight (g) × final body protein (%/dry basis)—initial body weight (g) × initial body protein (%/dry basis)]/total protein intake (g).

### 2.6. Sample Analysis

#### 2.6.1. Determination of Composition

The composition [crude protein (CP), crude lipid (CL), moisture (MS), and crude ash (CA)] of the diets and shrimp samples were determined using the AOAC method [22].

The amino acid composition of feed was determined following national standards GB/T 18246-2000 [23]. The samples were treated with a solution of hydrochloric acid at a concentration of 6 mol/L, frozen and charged with nitrogen, and then hydrolyzed at 110 °C for 22 h. Finally, the amino acid content was assayed with a fully automatic amino acid analyzer (model L8900, Hitachi, Tokyo, Japan).

#### 2.6.2. Chemical Analyses

Glutathione peroxidase (GSH-Px), total antioxidant capacity (T-AOC), and superoxide dismutase (SOD) were measured with Zhu et al. [24]. Glutamic-oxaloacetic transaminase (GOT) and glutamic-pyruvic transaminase (GPT) were determined following the method from Zhu et al. [25]. Acid phosphatase (ACP) and alkaline phosphatase (AKP) activities were assayed according to Xu [26]. Amylase (AMS), trypsin (TYS), and lipase (LPS) were measured by Li et al. [1]. Malonaldehyde (MDA) content and phenol oxidase (PO) activity were assayed with reference to An et al. [27]. Catalase (CAT) and lysozyme (LZM) were measured with Liu et al. [28,29].

#### 2.6.3. Analysis of Intestinal Microbiota

DNA was extracted using the MN NucleoSpin 96 Soi kit (Qiagen, Düsseldorf, Germany). A PCR reaction using primer 338F/806R (F: ACTCCTACGGGAGGCAGCA; R: GGACTACHVGGGTWTCTAAT) to generate a small fragment of the bacterial 16S-rRNA gene was conducted. Purified PCR amplicons were measured using the Monarch DNA Gel Extraction Kit (Invitrogen, USA). Sequences were performed on the Illumina MiSeq 2500 platform (Illumina, San Diego, CA, USA). After filtering the data and removing the chimeric sequences, the obtained sequences were gathered by operational classification units.

The datasets presented in this study can be found in online repositories. The names of the repository/repositories and accession number(s) can be found at: https://www.ncbi.nlm.nih.gov/bioproject/?term=PRJNA904075 (accessed on 10 December 2022).

#### 2.6.4. Real-Time PCR Analysis

Total RNA was obtained using an RNA extraction kit (TransZol Up Plus RNA Kit, Beijing, China). The PCR primers are presented in Table 3. A PrimeScript™ RT-PCR kit (TaKaRa, Kusatsu, Japan) was employed to synthesize complementary DNA (cDNA). PCR cycles referenced to Luo et al. [30]. All real-time PCR reactions were undertaken on a Roche LightCycler 480II (Basel, Switzerland) working with the SYBR @ Premix Ex TaqTMKit (Takara). Relative mRNA expression counted via the 2^−ΔΔCT^ method.

### 2.7. Statistical Analysis

All data were analyzed by one-way ANOVA with SPSS 21.0 (SPSS Inc., Chicago, IL, USA). Duncan’s multiple range test was performed if significant differences existed between groups (*p* < 0.05).

## 3. Results

### 3.1. Effects of RPM on Growth Performance

No significant differences in WG and FCR were found between the R10 and FM groups (*p* > 0.05; Table 4). In the R20, R40, R60 and R80 groups, both WG and SGR were significantly lower than in the FM group (*p* < 0.05). FCR was opposite to WG. As the RPM increased, PER first increased gradually and then decreased, with the R10 group being significantly higher than the FM group (*p* < 0.05). The changes in PER and PDR were consistent with each other.

### 3.2. Effects of RPM on Body Composition

The CP level was significantly lower in the R40, R60, and R80 groups than in the FM group (*p* < 0.05; Table 5). The MS level was significantly higher in the R40 groups than in the FM group (*p* < 0.05). No significant differences in CL and CA (*p* > 0.05) appeared.

### 3.3. Effects of RPM on Antioxidant Indexes

SOD activity was significantly higher in the R10 and R20 groups than in the FM group (*p* < 0.05; Table 6). The changes in GSH-Px activity and SOD activity were consistent with each other. CAT activity in the R10, R20, and R40 groups was significantly higher than that in the FM group (*p* < 0.05). T-AOC activity in the R10 and R20 groups was significantly higher than that in the FM group, whereas the opposite was observed for malondialdehyde (MDA) content (*p* < 0.05).

### 3.4. Effects of RPM on Non-Specific Immune Enzyme Activities

ACP and LZM activities were significantly higher in the R10, R20, and R40 groups than in the FM group (*p* < 0.05; Table 7). The AKP and PO activities in the R10 and R20 groups were significantly higher than that of those in the FM group (*p* < 0.05).

### 3.5. Effects of RPM on Digestive and Metabolic Enzyme Activities

GOT activity in the R10 group was significantly higher than those in the FM group (*p* < 0.05; Table 8). TYS and GPT activities were significantly higher in the R10 group than in the FM group, with the opposite being observed in the R60 and R80 groups (*p* < 0.05). No significant difference in AMS activity was found in R10, R20 and R40 groups compared to FM group (*p* > 0.05).

### 3.6. Effects of RPM on Hepatopancreatic Protein Synthesis Relative mRNA Expression

**Figure 1:**
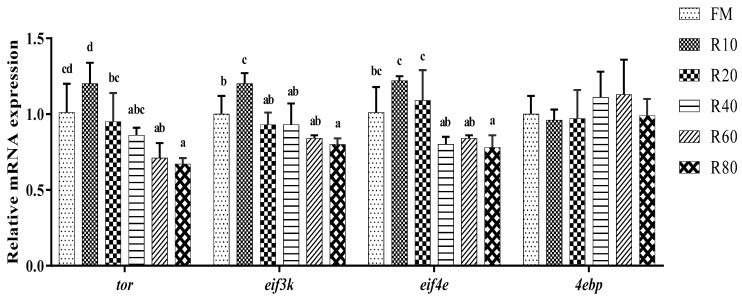
Effects of RPM replacing FM on hepatopancreatic protein synthesis relative mRNA expression for juvenile shrimp *L. vannamei*. Data are expressed as means ± SD (*n* = 3). Different letters above a bar were statistically significant different among treatments (*p* < 0.05).

No significant differences in *tor* mRNA levels were found among the R10, R20, R40, and FM groups (*p* > 0.05; Figure 1). The *eif3k* gene was significantly up-regulated in the R10 group and significantly down-regulated in the R80 group compared to the FM group (*p* < 0.05). The *eif4e* gene was significantly down-regulated in the R80 group compared to the FM group (*p* < 0.05). No significant differences in eukaryotic translation initiation factor 4E (*eif4e*) mRNA levels were observed among the R10, R20, R40, R60, and FM groups (*p* > 0.05).

### 3.7. Effects of RPM on Hepatopancreatic Non-Specific Immune Relative mRNA Expression

**Figure 2:**
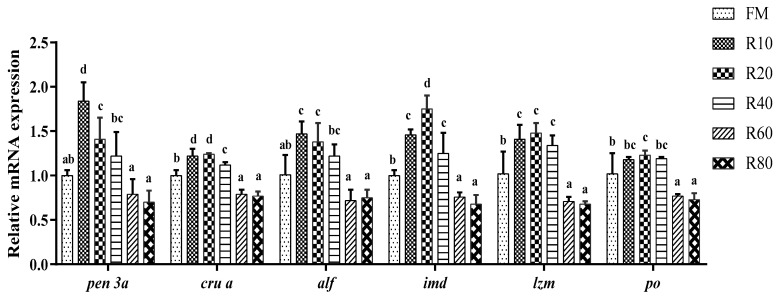
Effects of RPM replacing fishmeal on hepatopancreatic non-specific immune relative mRNA expression for juvenile shrimp *L. vannamei*. Data are expressed as means ± SD (*n* = 3). Different letters above a bar were statistically significant different among treatments (*p* < 0.05).

The penaiedin 3a (*pen 3a*) gene was significantly up-regulated in the R10 group compared to the FM group (*p* < 0.05; Figure 2). A significantly higher level of mRNA for crustin a (*cru a*), immunodeficiency (*imd*)*,* and lysozyme (*lzm*) were found in the R10, R20, and R40 groups than in the other groups (*p* < 0.05). The anti-lipopolysaccharide factor (*alf*) gene was significantly up-regulated in the R10 and R20 groups compared with those in the FM group (*p* < 0.05). The prophenoloxidase (po) mRNA level was significantly higher in the R20 group than in the FM group (*p* < 0.05).

### 3.8. Effects of RPM on the Intestinal Bacterial Community

For diversity indices (Table 9), the coverage of all samples was as high as 0.9987, indicating sufficient sequencing depth. The Ace and Chao1 indices were significantly higher in the R10 group than in the FM group (*p* < 0.05). The OTUs, Simpson, Shannon, and coverage indices were not significantly affected by the experimental diet (*p* > 0.05).

**Figure 3:**
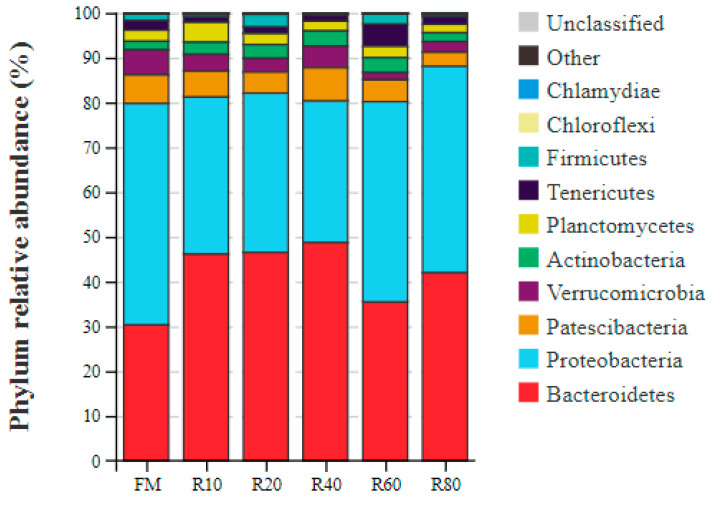
Composition of the intestinal microflora for juvenile shrimp *L. vannamei* at phylum level.

**Figure 4:**
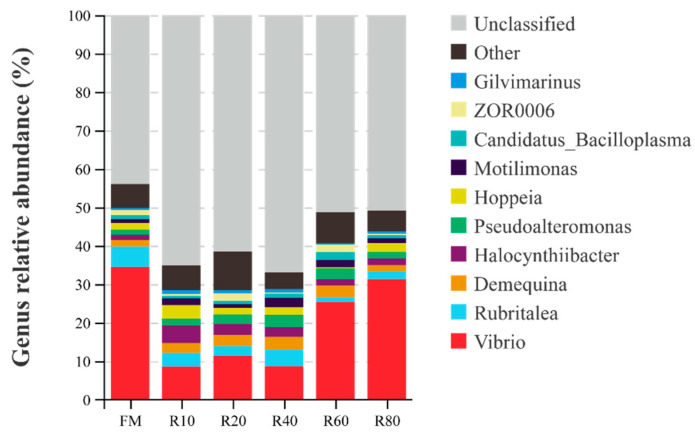
Composition of the intestinal microflora for juvenile shrimp *L. vannamei* at genus level.

The phyla with the top 10 highest relative abundance in juvenile *L. vannamei* intestinal microbial communities were Bacteroidetes, Proteobacteria, Patescibacteria, Patescibacteria, Actinobacteria, Actinobacteria, Tenericutes, Firmicutes, Chloroflexi, and Chlamydiae (Figure 3). Bacteroidetes and Proteobacteria abundances were over 80%. With the increase in the amount of RPM as FM replacement, the abundance of Bacteroidetes showed a trend of first increasing and then decreasing and was higher in all the replacement groups than in the FM group. Meanwhile, Proteobacteria showed an initially decreasing trend, followed by an increasing trend in relative abundance. The top 10 most abundant bacterial genera in the intestine were *Vibrio*, *Rubritalea*, *Demeqina*, *Halocynthiibacter*, *Pseudoalteromnas*, *Hoppeia*, *Motilimonas*, *Candidatus*, *Bacilloplasma*, ZOR0006, and *Gilvimarinus* (Figure 4). With the increase in the amount of RPM as FM replacement, the *Vibrio* relative abundance showed a decreasing and then increasing trend, with all replacement groups being lower than the FM group.

### 3.9. Effects of RPM on Challenge Test with V. parahaemolyticus

**Figure 5:**
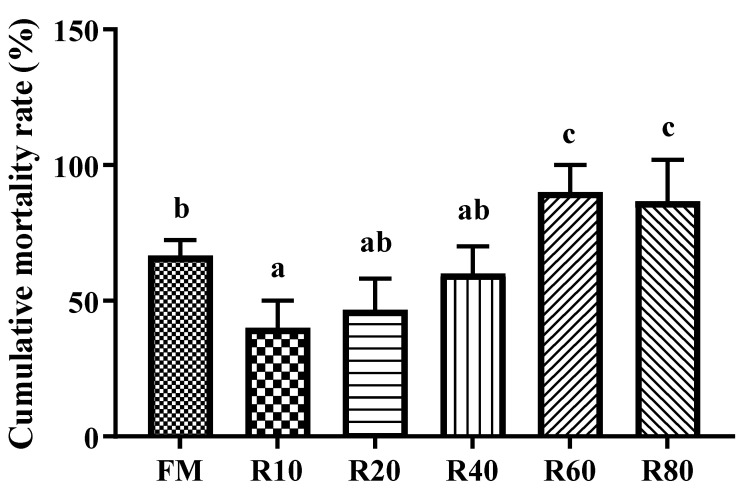
Cumulative mortality rate for juvenile shrimp *L. vannamei* challenged by *V. parahaemolyticus*. Data are expressed as means ± SD (*n* = 3). Different letters above a bar are statistically significant different among treatments (*p* < 0.05).

Figure 5 shows that, after the challenge test of *V. parahaemolyticus*, cumulative mortality was significantly lower in the R10 group compared to the FM group, with the opposite being observed in the R60 and R80 groups (*p* < 0.05).

## 4. Discussion

RPM is a high-energy, high-protein feed material with high amino acid content and good palatability [8]. At present, effects of RPM replacing FM on growth for shrimps have been reported. A study has shown that it is possible to replace 60% of FM (the base FM content: 30%) in juvenile *L. vannamei* (initial weight: 0.65 ± 0.01 g) feed with RPM based on indicators of growth and feed utilization [13]. In addition, Oujifard [14] revealed that RPM might be a potential candidate for 50% replacement of FM (the base FM content: 45.7%) in shrimp (Initial weight: 6.99 ± 0.08 g) feed. However, in the present study, RPM replacement for 10% FM (the base FM content: 20%) protein in shrimp (initial weight: 0.54 ± 0.01 g) feed showed no significant effects on WG, SGR, and FCR. When the proportion of RPM replacement increased, the WG of shrimp decreased significantly. This result is consistent with the variation in CP content of the shrimps. The current findings were consistent with the report of Oujifard [14]. In shrimp feed, a difference in the maximum percentage of RPM replacement was found between the present work and the above studies. The reasons for this discrepancy might be related to the quality of the RPM [32], the base FM content, the size of the shrimp [33], and other factors. Nevertheless, all of the above studies indicated that RPM could partially replace FM protein in juvenile *L. vannamei* feed.

Digestive enzyme activities reflect the most basic physiological characteristics of an animal’s digestion and its ability to utilize feed nutrients [34]. The growth of shrimps is related to their digestive enzyme activity. In the present study, replacing small amounts (10%) of FM with RPM increased the AMS and TYS activities. A previous work showed that the enzymatic digestion of rice protein produces a flavor peptide that replaces monosodium glutamate, effectively masking the bitterness and enhancing the viscosity and palatability of the feed [35]. Therefore, replacing small amounts of FM with RPM could improve digestive enzyme activities, thus ensuring that the shrimp could effectively absorb and digest the nutrients.

Animals grow by increasing the volume of tissue structures and accumulating protein; therefore, protein metabolism is often used as a measure of animal growth. Protein metabolism is mainly carried out in the hepatopancreas of shrimps through transamination and deamination. GPT and GOT are two of the most important transaminases in protein metabolism. GPT mainly plays an amino transfer role in catalyzing the reaction between α-ketoglutarate and aspartic acid to produce glutamic acid and oxaloacetic acid. GOT enables the transfer of amino acids between alanine and glutamate and regulates the synthesis of non-essential amino acids and the proteolytic processes. In the present study, replacing 10% FM with RPM significantly enhanced GPT and GOT activities, indicating that amino acid metabolism was accelerated, protein catabolism was decreased, and anabolism was enhanced. As a result, nitrogen accumulated in the shrimp body. The GPT and GOT activities decreased significantly when the proportion of RPM replacement was increased; the same trend was observed in *Megalobrama amblycephala* [36]. This phenomenon might be attributed to the relatively low lysine content of rice protein, resulting in an imbalance in the amino acid composition of the feed and thus affecting the metabolic homeostasis of amino acids in the liver.

The growth of shrimps is mainly achieved through protein synthesis. *Tor* is a highly conserved serine/threonine kinase, and its signaling pathway is vital for organisms to respond effectively to changes in their nutritional environment for regulating growth. *Eif4e*, a signaling factor downstream of the mammalian target of rapamycin (mTOR) signaling pathway, receives signals from TOR proteins through eIF4E-BP (*eif4e* binding protein) and regulates intracellular protein synthesis [37]. *Eif3k* acts as a structural protein and is involved in protein interactions [38]. In this study, the replacement of small amounts (10%) of FM with RPM increased *tor*, *eif3k*, and *eif4e* mRNA expression levels, indicating that RPM activated the mTOR pathway. An activated mTOR inhibits *4ebp1* phosphorylation, leading to the release of *4ebp1* from *eif4e*, reduced inhibition of *eif4e*-initiated translation by *4ebp1*, and increased expression of *eif4e* and thereby promoting the translation of key proteins associated with cell growth [39]. Under the present experimental conditions, the high protein content, rapid energy conversion, high digestibility, and good palatability of RPM allowed it to replace small amounts of FM in shrimp feed without significantly reducing the WGR.

SOD and CAT scavenge excess radicals from the body and decrease any oxidative damage to cells [40]. MDA is a peroxidation product and its concentration indicates the damage to the liver. GSH-Px assists in maintaining normal immune system function and has antioxidant and detoxifying effects [41]. T-AOC is a composite indicator of antioxidant capacity [42]. Rice protein has endogenous antioxidant effects and could effectively reduce oxidative stress damage to the body [43]. In the present study, the replacement of small amounts (10% and 20%) of FM with RPM significantly increased the SOD, CAT, GSH-Px, and T-AOC activities and reduced the MDA content. A previous work found that RPM was decomposed by a protease to form different bioactive peptides, and the scavenging rate of 1,1-diphenyl-2-picrylhydrazyl free radicals reached 54.5% [44]. In the present research, the replacement of a small amount (10%) of FM with RPM significantly increased the TYS activity and aided the digestion of RPM by shrimp. A study showed that rice proteins could be involved in the Nrf2-ARE signaling pathway, thus regulating antioxidant enzyme expression [45,46]. However, Fu’s study [13] found no significant differences in the SOD activity and MDA content of shrimps fed with partial RPM replacement. We speculate that the processing of rice proteins can influence their physico-chemical and structural properties, as well as their structure, solubility, and hydrolytic capacity [47,48,49]. Therefore, under the present experimental conditions, the replacement of a small amount of FM with RPM significantly increased the antioxidant capacity of the shrimps.

The non-specific immune system is the sole defense of invertebrates against pathogenic invasion [50]. PO, AKP, and ACP are vital regulatory enzymes and are essential in the non-specific immune response of crustaceans [51]. LZM has the function of hydrolyzing and digesting invading pathogens and inducing the synthesis and production of related immune factors [52]. In the present study, replacing 10% of FM with RPM significantly increased PO, AKP, ACP, and LZM activities, in line with Fu’s findings [13]. Moreover, the replacement of small amounts of FM with RPM increased *lzm* and *po* mRNA expression. Previously, a study showed that the enzymatic digestion of RPM by trypsin produces a bioactive peptide, oryzatensin, which has immunomodulatory activity [53].

The intestinal bacterial flora, an important micro-ecosystem, relates to digestion, absorption, and immune function [54]. Under normal conditions, a balance between the various bacteria in the gut occurs dynamically to maintain the stability of the intestinal environment, thus contributing to the effective suppression of invasion by exogenous pathogenic bacteria and the enhancement of nonspecific immunity [55]. Richness and diversity indices are important indicators of microbial diversity and complexity, and differences in their values reflect variations in community structure and numbers of species [56]. Ace and Chao1 indices are commonly used to calculate colony richness, with high values indicating a large number of species in the sample. Meanwhile, the Shannon and Simpson indices are applied to calculate colony diversity, with high values indicating high species diversity [57]. In the present study, Ace and Chao1 values in the R10 group were significantly higher than those in the control group, and no significant difference in Simpson and Shannon values were observed. This finding indicates that the replacement of a small amount of FM with RPM in the diet increased the species richness of the shrimp gut microbiota without altering its diversity.

At the phylum level, the main bacterial groups in the gut of shrimp were the phyla Bacteroidetes and Proteobacteria [16]. Similar conclusions were reached in the present study, with Bacteroidetes and Proteobacteria abundances at over 80%. Bacteroidetes help the host to digest proteins, carbohydrates (especially polysaccharides), and other substances to increase the availability of nutrients; these bacteria are also involved in the metabolism and transport of sugars, provide energy for the host and thus promote its growth [58]. Proteobacteria are highly correlated with sample spoilage, and their relative proportion in the gut of diseased animals is significantly elevated [59]. In the present study, the replacement of a small amount (10–40%) of FM with RPM significantly increased Bacteroidetes abundance but reduced that of Proteobacteria. At the genus level, *Vibrio* abundance decreased significantly and then increased with increasing RPM replacement amount. *Vibrio* is the dominant genus in the sea and one of the main pathogenic bacteria for mariculture animals [60]. Therefore, the replacement of small amounts (10–40%) of FM with RPM could improve the microbiological composition of the shrimp gut.

The hepatopancreas is the main immune organ of the shrimp, and the innate immunity is an important defense for invertebrates against disease-causing agents. A range of immune factors, such as *penaiedin 3a*, *lzm*, *alf*, and *crustin*, play a vital function in humoral immunity and are closely linked to the strength of resistance against infection in *L. vannamei*. In invertebrates, the innate immunodeficiency gene IMD encodes an immunodeficiency protein, IMD. The IMD protein transmits the signal of an invading specific foreign body or pathogenic bacteria from outside the cell to inside the cell and causes a series of cascade responses in the cell to form immune effectors (e.g., antimicrobial peptides) [61]. In this study, the replacement of a small amount of FM with RPM significantly up-regulated the expression of *pen 3a*, *cru a*, *alf*, *imd*, and *lzm*. *V. parahaemolyticus* is a Gram-negative bacterium that is highly pathogenic to shrimps such as *L. vannamei*, *Fenneropenaeus chinensis*, and *Marsupenaeus japonicus* [62]. In this work, an attack experiment using *V. parahaemolyticus* showed the lowest cumulative mortality in the 10% substitution group. Meanwhile, a 60% substitution rate resulted in a significant increase in cumulative mortality in shrimps. Takahashi [53] showed that the hydrolysis of rice proteins by trypsin yields the immunocompetent nine-band peptide Gly-Tyr-Pro-Met-Tyr-Pro-Leu-Pro-Arg, which enhances the phagocytosis of leukocytes. Therefore, replacing FM with moderate amounts of RPM could significantly improve the immune regulation and resistance to diseases of shrimp.

## 5. Conclusions

RPM can be reasonably used to replace a small amount of FM in shrimp feed. No significant effect on shrimp growth was found with partial RPM replacement of FM (10%), but digestibility, protein synthesis, antioxidant capacity, non-specific immunity, and disease resistance were significantly improved. The replacement of 10–40% with RPM for FM improved the intestinal flora structure of the shrimp.

## Figures and Tables

**Table 1:** Formula and nutritional composition of diets (g·kg^−1^).

Ingredients	Groups					
	FM	R10	R20	R40	R60	R80
Brown fish meal	200.00	180.00	160.00	120.00	80.00	40.00
Rice protein meal	0.00	18.00	36.10	72.10	108.20	144.20
Soybean meal	150.00	150.00	150.00	150.00	150.00	150.00
Peanut meal	90.00	90.00	90.00	90.00	90.00	90.00
Corn gluten meal	60.00	60.00	60.00	60.00	60.00	60.00
Beer yeast	50.00	50.00	50.00	50.00	50.00	50.00
Shrimp shell powder	60.00	60.00	60.00	60.00	60.00	60.00
Wheat flour	230.00	230.00	230.00	230.00	230.00	230.00
Ca(H_2_PO_4_)_2_	15.00	15.00	15.00	15.00	15.00	15.00
Vitamin C	0.30	0.30	0.30	0.30	0.30	0.30
Choline chloride	0.50	0.50	0.50	0.50	0.50	0.50
Soybean lecithin + Soybean oil + Fish oil ^1^	25.00	26.40	27.80	30.60	33.40	36.20
Vitamin and mineral premix ^2^	10.0	10.0	10.0	10.0	10.0	10.0
DL-Methionine	1.70	1.80	1.90	2.20	2.40	2.60
L-Lysine	1.70	2.50	3.30	4.80	6.40	7.90
L-Threonine	0.00	0.10	0.20	0.50	0.70	1.00
Microcrystalline cellulose	105.80	105.40	104.90	104.00	103.10	102.30
Total	1000.00	1000.00	1000.00	1000.00	1000.00	1000.00
**Nutritional composition ^3^**						
Crude protein (%)	38.09	37.58	38.07	37.73	37.89	37.98
Crude lipid (%)	7.72	7.60	7.88	7.96	7.58	7.25
Crude ash (%)	9.08	9.21	9.12	8.95	8.84	8.91
Moisture (%)	8.27	8.81	8.42	8.71	8.50	8.61
Carbohydrates (%)	26.32	26.01	25.62	25.03	24.68	24.41
Digestible energy (kJ/g)	12.72	12.48	12.60	12.34	12.02	11.82

^1^ The proportions of soybean lecithin, soybean oil and fish oil are 3:1:1, respectively. ^2^ Vitamin and mineral premix (per kg): vitamin A, 5000 IU; thiamine, 5 mg; riboflavin, 10 mg; vitamin C, 150 mg; vitamin D3, 1000 IU; vitamin E, 40 mg; menadione, 10 mg; pyridoxine, 10 mg; biotin, 0.1 mg; cyanocobalamin, 0.02 mg; calcium pantothenate, 20 mg; folic acid, 1 mg; niacin, 40 mg; iron, 100 mg; iodine, 0.8 mg; cupper, 3 mg; zinc, 50 mg; manganese, 12 mg; selenium, 0.3 mg; cobalt, 0.2 mg. ^3^ Crude protein, crude lipid, crude ash, carbohydrates, and moisture contents are measured values. Digestible energy is a calculated value.

**Table 2:** Amino acid composition of diet.

Amino Acid	Groups (% Dry Matter)					
	FM	R10	R20	R40	R60	R80
Asp ^#^	3.68	3.53	3.58	3.69	3.56	3.64
Thr *	1.48	1.45	1.44	1.43	1.49	1.52
Ser ^#^	1.69	1.73	1.75	1.73	1.74	1.77
Glu ^#^	6.99	6.91	6.90	6.88	6.88	6.82
Gly ^#^	2.01	1.96	1.98	2.03	2.01	2.07
Ala ^#^	2.16	2.07	2.12	2.11	2.02	2.10
Cys ^#^	0.45	0.44	0.46	0.53	0.49	0.44
Val *	1.79	1.73	1.78	1.77	1.85	1.75
Met *	0.76	0.75	0.82	0.76	0.63	0.65
Ile *	1.55	1.49	1.57	1.52	1.45	1.63
Leu *	3.12	3.06	3.07	3.11	3.01	3.00
Tyr ^#^	1.23	1.21	1.24	1.20	1.06	1.12
Phe *	1.81	1.75	1.78	1.75	1.85	1.81
Lys *	2.33	2.27	2.35	2.22	2.29	2.35
His *	1.06	1.00	1.03	0.97	1.08	0.96
Arg *	2.50	2.40	2.47	2.40	2.44	2.29
Pro ^#^	2.05	2.17	2.03	2.08	2.10	2.01
TAA	36.65	35.92	36.37	36.19	35.97	35.91

* Essential amino acids; ^#^ non-essential amino acids. Asp: aspartate; Thr: threonine; Ser: serine; Glu: glutamate; Gly: glycine; Ala: alanine; Cys: cystine; Val: valine; Met: methionine; Ile: isoleucine; Leu: leucine; Tyr: tyrosine; Phe: phenylalanine; Lys: lysine; His: histidine; Arg: arginine; Pro: proline; TAA: total amino acids.

**Table 3:** Primers pair sequences used in real-time PCR.

Primer	Sequences (5’ to 3’)	Source or GenBank
*β-actin*	F: TCACCGTCTCCATCACCTGC	AF300705.2
	R: GAAGCCAATACCTTGTCATACTCC	
*pen 3a*	F: ATACCCAGGCCACCACCCTT	DQ206403.1
	R: TGACAGCAACGCCCTAACC	
*cru a*	F: GGTGTTGGTGGTGGTTTCCC	AY486426.1
	R: CAGTCGCTTGTGCCAGTTCC	
*alf*	F: CGCTTCACCGTCAAACCTTAC	GQ227486.1
	R: GCCACCGCTTAGCATCTTGTT	
*imd*	F: CGGCTCTGCGGTTCACAT	FJ592176
	R: CCTCGACCTTGTCTCGTTCCT	
*lzm*	F: TGTTCCGATCTGATGTCC	AY170126.2
	R: GCTGTTGTAAGCCACCC	
*po*	F: GCCTTGGCAACGCTTTCA	EF115296.1
	R: CGCGCATCAGTTCAGTTTGT	
*tor*	F: TGCCAACGGGTGGTAGA	MN398907.1
	R: GGGTGTTTGTGGACGGA	
*4ebp*	F: ATGTCTGCTTCGCCCGTCGCTCGCC	XM_027367939.1
	R: GGTTCTTGGGTGGGCTCTT	
*eif4e*	F: TGGAATCAAACCTATGTGGG	Xie et al. [31]
	R: GTCCTCCTGGAAGCGTA	
*eif3k*	F: GCGAGAACACCTATGACC	DQ398574.1
	R: GAGGCACTTGGACAGAAC	

Note: *pen 3a*: penaiedin 3a; *cru a*: crustin a; *alf*: anti-lipopolysaccharide factor; *imd*: immune deficiency; *lzm*: lysozyme; *po*: prophenoloxidase; *tor*: target of rapamycin; *4ebp*: 4E-binding protein; *eif4e*: eukaryotic translation initiation factor 4E; *eif3k*: eukaryotic translation initiation factor 3K.

**Table 4:** Effects of RPM replacing fishmeal on growth for *L. vannamei*.

Items	Groups					
	FM	R10	R20	R40	R60	R80
Initial weight	0.55 ± 0.01	0.55 ± 0.01	0.54 ± 0.01	0.54 ± 0.01	0.54 ± 0.01	0.54 ± 0.01
Final weight	6.89 ± 0.10 ^c^	7.04 ± 0.21 ^c^	6.39 ± 0.15 ^b^	6.18 ± 0.05 ^ab^	6.07 ± 0.06 ^a^	5.97 ± 0.10 ^a^
WG (%)	1163.82 ± 20.82 ^c^	1189.86 ± 42.41 ^c^	1074.93 ± 30.57 ^b^	1036.19 ± 12.14 ^ab^	1015.69 ± 5.57 ^a^	1001.29 ± 23.32 ^a^
SGR (%/d)	4.70 ± 0.03 ^c^	4.73 ± 0.06 ^c^	4.56 ± 0.05 ^b^	4.50 ± 0.02 ^ab^	4.47 ± 0.01 ^a^	4.44 ± 0.04 ^a^
FCR	1.35 ± 0.02 ^a^	1.32 ± 0.02 ^a^	1.46 ± 0.01 ^b^	1.49 ± 0.02 ^bc^	1.52 ± 0.02 ^c^	1.58 ± 0.04 ^d^
SR (%)	95.83 ± 2.89	95.83 ± 2.89	97.5 ± 2.50	98.33 ± 1.44	98.33 ± 1.44	96.67 ± 2.89
PER	1.94 ± 0.02 ^d^	2.01 ± 0.04 ^e^	1.80 ± 0.01 ^c^	1.77 ± 0.02 ^c^	1.73 ± 0.02 ^b^	1.66 ± 0.04 ^a^
PDR (%)	37.66 ± 0.38 ^d^	39.77 ± 1.57 ^e^	34.15 ± 0.04 ^c^	32.26 ± 0.45 ^b^	32.55 ± 1.06 ^b^	30.33 ± 0.94 ^a^

Note: Data are mean ± *SD* (*n* = 3). Values in the same row with different superscripts represent significant differences (*p* < 0.05).

**Table 5:** Effects of RPM replacing fishmeal on whole body composition for *L. vannamei*.

Items	Groups (% Dry Matter)					
	FM	R10	R20	R40	R60	R80
CP (%)	72.72 ± 0.96 ^cd^	74.08 ± 1.00 ^d^	71.9 ± 1.54 ^bc^	70.52 ± 1.39 ^ab^	69.42 ± 0.21 ^a^	69.11 ± 0.41 ^a^
CL (%)	8.31 ± 0.50	9.1 ± 0.93	9.36 ± 0.75	8.78 ± 0.70	8.4 ± 0.48	8.18 ± 0.87
CA (%)	12.89 ± 0.49	11.76 ± 0.75	12.2 ± 1.42	12.41 ± 0.98	12.21 ± 0.77	12.06 ± 0.67
MS (%)	75.47 ± 0.17 ^a^	75.45 ± 0.31 ^a^	75.91 ± 0.62 ^ab^	76.52 ± 0.36 ^b^	75.34 ± 0.71 ^ab^	76.09 ± 0.32 ^a^

Note: Data are mean ± *SD* (*n* = 3). Values in the same row with different superscripts represent significant differences (*p* < 0.05).

**Table 6:** Effects of RPM replacing fishmeal on serum antioxidant indexes for *L. vannamei*.

Items	Groups					
	FM	R10	R20	R40	R60	R80
SOD (U/mL)	814.68 ± 43.03 ^b^	1031.6 ± 64.62 ^c^	973.14 ± 68.82 ^c^	865.98 ± 61.73 ^b^	694.93 ± 37.57 ^a^	679.31 ± 37.45 ^a^
CAT (U/mL)	32.43 ± 0.75 ^a^	41.55 ± 1.88 ^bc^	45.45 ± 2.16 ^c^	38.42 ± 3.58 ^b^	30.42 ± 2.70 ^a^	27.31 ± 4.32 ^a^
GSH-Px (U/mL)	104.90 ± 7.95 ^b^	130.14 ± 17.23 ^c^	113.62 ± 13.36 ^bc^	95.72 ± 7.50 ^ab^	82.62 ± 3.43 ^a^	83.49 ± 4.67 ^a^
MDA (mmol/L)	12.92 ± 0.66 ^bc^	9.49 ± 2.25 ^a^	9.66 ± 1.08 ^a^	11.00 ± 0.39 ^ab^	13.81 ± 2.08 ^d^	14.14 ± 0.52 ^d^
T-AOC (mM)	0.40 ± 0.06 ^a^	0.59 ± 0.02 ^b^	0.55 ± 0.04 ^b^	0.43 ± 0.05 ^a^	0.36 ± 0.03 ^a^	0.35 ± 0.04 ^a^

Note: Data are mean ± *SD* (*n* = 3). Values in the same row with different superscripts represent significant difference (*p* < 0.05).

**Table 7:** Effects of RPM replacing fishmeal on serum non-specific immune enzyme activities for *L. vannamei*.

Items	Groups					
	FM	R10	R20	R40	R60	R80
ACP (U/L)	68.03 ± 8.40 ^b^	86.02 ± 5.71 ^c^	83.77 ± 7.04 ^c^	78.71 ± 2.45 ^c^	50.79 ± 4.22 ^a^	50.22 ± 5.63 ^a^
AKP (U/L)	28.45 ± 8.09 ^ab^	43.66 ± 3.99 ^c^	30.17 ± 6.71 ^ab^	28.86 ± 3.10 ^ab^	25.30 ± 2.45 ^ab^	19.86 ± 3.10 ^a^
PO (U/L)	22.03 ± 4.46 ^ab^	30.57 ± 2.82 ^c^	26.7 ± 2.65 ^bc^	22.36 ± 2.74 ^ab^	16.71 ± 2.97 ^a^	17.15 ± 1.47 ^a^
LZM (U/L)	4.00 ± 0.09 ^b^	5.53 ± 0.43 ^c^	5.13 ± 0.14 ^c^	5.20 ± 0.34 ^c^	3.66 ± 0.22 ^ab^	3.46 ± 0.11 ^a^

Note: Data are mean ± *SD* (*n* = 3). Values in the same row with different superscripts represent significant differences (*p* < 0.05).

**Table 8:** Effects of RPP replacing fishmeal on hepatopancreas digestive and metabolic enzyme activities for *L. vannamei*.

Items	Groups					
	FM	R10	R20	R40	R60	R80
AMS (U/mg)	3.74 ± 0.59 ^cd^	4.38 ± 0.27 ^d^	4.15 ± 0.41 ^d^	3.46 ± 0.33 ^bc^	2.97 ± 0.26 ^ab^	2.66 ± 0.10 ^a^
TYS (U/mg)	27.92 ± 5.72 ^b^	33.08 ± 2.39 ^c^	27.74 ± 0.96 ^b^	24.00 ± 1.83 ^ab^	21.46 ± 0.69 ^a^	20.78 ± 1.75 ^a^
LPS (U/g)	16.58 ± 1.63	17.44 ± 1.47	18.68 ± 2.46	18.33 ± 1.72	18.47 ± 0.77	17.20 ± 0.36
GPT (U/g)	29.84 ± 5.17 ^b^	37.56 ± 3.06 ^c^	30.32 ± 5.27 ^b^	24.9 ± 2.94 ^ab^	20.09 ± 0.7 ^a^	19.58 ± 4.67 ^a^
GOT (U/g)	68.77 ± 5.86 ^ab^	83.12 ± 4.50 ^c^	70.78 ± 5.86 ^bc^	65.98 ± 11.19 ^ab^	57.47 ± 6.73 ^ab^	56.14 ± 7.22 ^a^

Note: Data are mean ± *SD* (*n* = 3). Values in the same row with different superscripts represent significant differences (*p* < 0.05).

**Table 9:** Effects of RPM replacing fishmeal on intestinal species diversity for *L. vannamei*.

Items	Groups					
	FM	R10	R20	R40	R60	R80
OTUs	442.33 ± 72.80	478.67 ± 26.10	466.67 ± 13.05	451.33 ± 43.15	432.33 ± 33.53	467.00 ± 19.00
Ace	492.22 ± 66.27 ^a^	582.03 ± 18.52 ^b^	541.35 ± 44.78 ^ab^	532.98 ± 52.45 ^ab^	481.04 ± 43.69 ^a^	543.8 ± 18.24 ^ab^
Chao1	478.2 ± 63.51 ^a^	563.69 ± 11.27 ^b^	526.38 ± 38.58 ^ab^	509.6 ± 44.75 ^ab^	485.56 ± 46.19 ^ab^	541.39 ± 1.73 ^ab^
Simpson	0.86 ± 0.03	0.86 ± 0.04	0.89 ± 0.04	0.84 ± 0.02	0.89 ± 0.04	0.84 ± 0.02
Shannon	4.13 ± 0.26 ^ab^	4.36 ± 0.24 ^ab^	4.72 ± 0.70 ^b^	3.97 ± 0.15 ^a^	4.52 ± 0.32 ^ab^	4.01 ± 0.12 ^a^
Coverage	0.9992 ± 0.0002	0.9987 ± 0.0001	0.999 ± 0.0005	0.9989 ± 0.0002	0.9993 ± 0.0002	0.999 ± 0.0002

Note: Data are mean ± *SD* (*n* = 3). Values in the same row with different superscripts represent significant differences (*p* < 0.05).

## Data Availability

Data supporting the results of this study are provided on request to the respective authors. This data is not disclosed due to privacy or ethical constraints.

## References

[1] Li W., Li L., Liu H., Tan B., Dong X., Yang Q., Chi S., Zhang S., Xie R. (2022). Effects of clostridium butyricum on growth, antioxidant capacity and non-specific immunology of *Litopenaeus vannamei* fed with concentrated cottonseed protein replacement of fishmeal. J. Guangdong Ocean. Univ..

[2] Wang J., Liang D., Yang Q., Tan B., Dong X., Chi S., Liu H., Zhang S. (2020). The effect of partial replacement of fish meal by soy protein concentrate on growth performance, immune responses, gut morphology and intestinal inflammation for juvenile hybrid grouper (*Epinephelus fuscoguttatus* female × *Epinephelus lanceolatus* male). Fish Shellfish. Immunol..

[3] Gyan W.R., Yang Q., Tan B., Jan S.S., Jiang L., Chi S., Dong X., Liu H., Shuang Z. (2020). Effects of antimicrobial peptides on growth, feed utilization, serum biochemical indices and disease resistance of juvenile shrimp, *Litopenaeus vannamei*. Aquac. Res..

[4] Lin H., Tan B., Ray G.W., Zeng M., Li M., Chi S., Yang Q. (2021). A challenge to conventional fishmeal: Effects of soy protein peptides on growth, histomorphology, lipid metabolism and intestinal health for juvenile Pompano *Trachinotus ovatus*. Front. Mar. Sci..

[5] Ray G.W., Liang D., Yang Q., Tan B., Dong X., Chi S., Liu H., Zhang S., Rimei L. (2020). Effects of replacing fishmeal with dietary soybean protein concentrate (SPC) on growth, serum biochemical indices, and antioxidative functions for juvenile shrimp *Litopenaeus vannamei*. Aquaculture.

[6] Lin H., Yang Q., Wang A., Wang J., Tan B., Ray G.W., Dong X., Chi S., Liu H., Zhang S. (2021). Effects of fish meal under different storage conditions on growth, serum biochemical indices and antioxidant capacity for juvenile grouper *Epinephelus coioides*. Aquac. Nutr..

[7] Zhu Z.H., Yang Q.H., Tan B.P., Zhou X.Q., Dong X.H., Chi S.Y., Liu H.Y., Zhang S. (2021). Effects of replacing fishmeal with soybean protein concentrate (SPC) on growth, blood biochemical indexes, non-specific immune enzyme activity, and nutrient apparent digestibility for juvenile *Litopenaeus vannamei*. Aquac. Int..

[8] Palmegiano G., Daprà F., Forneris G., Gai F., Gasco L., Guo K., Peiretti P., Sicuro B., Zoccarato I. (2006). Rice protein concentrate meal as a potential ingredient in practical diets for rainbow trout (Oncorhynchus mykiss). Aquaculture.

[9] Shih F.F., Daigle K.W. (2000). Preparation and characterization of rice protein isolates. J. Am. Oil Chem. Soc..

[10] Chen Y., Zhang Z.G. (2017). Research progress in rice protein. Grain Fats.

[11] Wani M.A., Tyagi P.K., Mir N.A., Hazarika R., Sheikh S.A., Tyagi P.K., Dinani O.P., Mandal A.B. (2018). Feeding value of rice gluten meal as an alternate protein source in broiler chickens. Turk. J. Veter-Anim. Sci..

[12] Sharif M.K., Butt M.S., Anjum F.M., Khan S.H. (2014). Rice Bran: A Novel Functional Ingredient. Crit. Rev. Food Sci. Nutr..

[13] Fu J.H., Deng M., Wen X.K., Liu S.L., Su Y.L., Guo C. (2017). Study on fish meal replaced by rice protein powder in pacific white shrimp *Litopenaeus vannamei*. China Feed..

[14] Oujifard A., Seyfabadi J., Kenari A.A., Rezaei M. (2012). Growth and apparent digestibility of nutrients, fatty acids and amino acids in Pacific white shrimp, *Litopenaeus vannamei*, fed diets with rice protein concentrate as total and partial replacement of fish meal. Aquaculture.

[15] Ray G.W., Yang Q., Tan B., Dong X., Chi S., Liu H., Zhang S. (2021). Effects of replacing fishmeal with dietary wheat gluten meal (WGM) on growth, serum biochemical indices, and antioxidative functions, gut microbiota, histology and disease resistance for juvenile shrimp *Litopenaeus vannamei*. Anim. Feed Sci. Technol..

[16] Wu Y.C., Li R.M., Shin G.R., Huang F., Yang Q.H., Tan B.P., Chi S.Y. (2021). Effects of dietary small peptides on growth, antioxidant capacity, nonspecific immunity and ingut microflora structure of *Litopenaeus vannamei*. J. Guangdong Ocean. Univ..

[17] He S., Ding M., Ray G.W., Yang Q., Tan B., Dong X., Chi S., Liu H., Zhang S. (2021). Effect of dietary vitamin D levels on growth, serum biochemical parameters, lipid metabolism enzyme activities, fatty acid synthase and hepatic lipase mRNA expression for orange-spotted grouper (*Epinephelus coioides*) in growth mid-stage. Aquac. Nutr..

[18] Wang A.J., Yang Q.H., Tan B.P., Xiao W.W., Jia J., Dong X.H., Chi S.Y., Liu H.Y., Zhang S. (2018). Effects of enzymolytic soybean meal on growth performance, serum biochemical indices, non-specific immunity and disease resistance of juvenile *Litopenaeus vannamei*. J. Guangdong Ocean. Univ..

[19] Zhang K., Liu S.C., Fan X.P., Wei S., Sun Q.X., Xia Q.Y., Ji H.W., Hao J.M., Deng C.J. (2021). Review on strategies and key technologies of live fish transportation. J. Guangdong Ocean. Univ..

[20] Lin H., Tan B., Yang Q., Chi S., Wei H., Wu Y., Ray G.W., Yohana M.A. (2022). Effects of dietary glycerol monolaurate on growth, antioxidant capacity and lipid metabolism in cage-farmed Pompano (*Trachinotus ovatus*) juveniles. Front. Mar. Sci..

[21] Xie J.H., Qiu D.Q., Liu C.X., Zhu W.W., Zeng L. (2013). Effcets of *vibrio alginolyticus* peptidoglycan on astaxanthin level, immune indicators and protection in *Litopenaeus vannamei*. J. Guangdong Ocean. Univ..

[22] Horwitz W. (2010). Official Methods of Analysis of AOAC International.

[23] Zhang W., Tan B.P., Pang A.B., Deng J.M., Yang Q.H., Zhang H.T. (2022). Screening of potential biomarkers for soybean meal induced enteritis in pearl gentian grouper, *Epinephelus fuscoguttatus* ♀× *Epinephelus lanceolatus* ♂. J. Guangdong Ocean. Univ..

[24] Zhu X.F., Deng Q.X., Guo H., Li G.L., Zhu C.H. (2021). Effect of hydrolyzable tannins on hemolymph and cellular immunological responses of *Litopenaeus vannamei* challenged by *Vibrio parahaemolyticus*. J. Guangdong Ocean. Univ..

[25] Zhu Q.G., Lin J.B., Huang C.J., Chen D.H., Liang P., Qin C.Q., Lin K.B. (2012). Effects of dietary n-3 highly unsaturated fatty acids on growth and Muscle fatty acid composition of juvenile grouper (*Epinephlus coioides*). J. Guangdong Ocean. Univ..

[26] Xu D.F., Wu J.X., Sun L.J., Qin S.M., Fan X.P. (2022). Physiological response and metabolic regulation of *Litopenaeus vannamei* exposed to combination stress of acute cold exposure and chronic waterless duration. J. Guangdong Ocean. Univ..

[27] An W.Q., Lai W.W., Tan B.P., Yang Q.H., Dong X.H., Liu H.Y., Chen X., Li W., Zhao X. (2018). Optimum calcium and phosphorus supplemental levels in diets of large size *Litopenaeus vannamei*. J. Guangdong Ocean. Univ..

[28] Liu H.Y., Li L.X., Ayiku A., Tang Z., Fan W., Tan B.P., Dong X., Chi S., Yang Q., Zhang S. (2021). Effects of dietary yeast culture supplementation on growth, intestinal morphology, immune, and disease resistance in *Epinephelus fuscoguttatus* ♀× *Epinephelus lanceolatu* ♂. J. Guangdong Ocean. Univ..

[29] Lu Z.F., Huang H., Huang X.M., Huang W.Z. (2022). Effects of hypoxic stress on antioxidant and energy metabolism of hybrid grouper (*Epinephelus fuscoguttatus* ♀ × *Epinephelus lanceolatus* ♂). J. Guangdong Ocean. Univ..

[30] Luo J., Fu W.J., Yang E.J., Huang J.S., Xie R.T., Chen G. (2022). Effects of quercetin on growth performance, antioxidant capacity and intestinal microflora of hybrid grouper (*Epinephelus fuscoguttatus*♀× *Epinephelus polyphekadion*♂). J. Guangdong Ocean. Univ..

[31] Xie S., Wei D., Yin P., Zheng L., Guo T., Liu Y., Tian L., Niu J. (2019). Dietary replacement of fish-meal impaired protein synthesis and immune response of juvenile Pacific white shrimp, *Litopenaeus vannamei* at low salinity. Comp. Biochem. Physiol. Part B Biochem. Mol. Biol..

[32] Sudaryono A., Tsvetnenko E., Evans L.H. (2001). Evaluation of potential of lupin meal as an alternative to fish meal in juvenile *Penaeus monodondiets*. Aquac. Nutr..

[33] Akiyama D.M. (1989). Soybean Meal Utilization by Marine Shrimp. Paper Presented at the Proceeding of the World Congress, Vegetable Protein Utilization in Human Foods and Animal Feedstuffs.

[34] Mousavi E., Mohammadiazarm H., Mousavi S.M., Ghatrami E.R. (2016). Effects of inulin, savory and onion powders in diet of juveniles carp *cyprinus carpio* (Linnaeus 1758) on gut micro flora, immune response and blood biochemical parameters. Turk. J. Fish. Aquat. Sci..

[35] Fang J.J., Liang Y., Lin P.R., Wu Y., Wu W., Gao Y. (2012). Functional effects, research methods and application prospects of rice active peptide. Food Ferment. Ind..

[36] Cai W.C., Li X.F., Jiang G.Z., Liu S.Q., Liu W.B. (2017). Effects of fish meal replacement by rice protein concentrate on growth, intestinal digestive and absorptive capability and amino acid metabolism of blunt snout bream (*Megalobrama amblycephala*). J. Nanjing Agric. Univ..

[37] Laplante M., Sabatini D.M. (2012). mTOR signaling in growth control and disease. Cell.

[38] Dong Z., Zhang J.T. (2006). Initiation factor eIF3 and regulation of mRNA translation, cell growth, and cancer. Crit. Rev. Oncol. Hematol..

[39] Hashemolhosseini S., Nagamine Y., Morley S.J., Desrivières S., Mercep L., Ferrari S. (1998). Rapamycin Inhibition of the G1 to S Transition Is Mediated by Effects on Cyclin D1 mRNA and Protein Stability. J. Biol. Chem..

[40] Sun Y., Jiang C., Li Y.Q., Mao M.G., Wu H., Gong X. (2013). Effects of dietary lipid on the liver antioxidant capacity and histology of *Takifugu rubripes* juvenile. J. Guangdong Ocean. Univ..

[41] Liang D., Zheng Q., Yang Q., Tan B., Dong X., Chi S., Liu H., Zhang S. (2021). Alterations on growth performance, antioxidant responses and lipid metabolism in liver for juvenile hybrid grouper (*Epinephelus fuscoguttatus* ♀ × *Epinephelus lanceolatus* ♂) fed dietary vitamin E. Aquac. Rep..

[42] Hu B., Song L.P., Mao S.Q., Xu P. (2019). Effects of four chinese herbal preparations on growth performance and antioxidant activity in Juvenile *Micropterus salmoides*. J. Guangdong Ocean. Univ..

[43] Yang L., Chen J.H., Lv J., Wu Q., Xu T., Zhang H., Liu Q.H., Yang H.K. (2012). Rice protein improves adiposity, body weight and reduces lipids level in rats through modification of triglyceride metabolism. Lipids Health Dis..

[44] Cao F., Ma H.L., Qu W.J., Jia J.Q., Pan Z.L., Ding Q.Z., Luo L., Wang Z.B., He R.H. (2009). Enzymatic hydrolysis of rice protein with papain and antioxidation activity of hydrolysate. J. Chin. Cereals Oils Assoc..

[45] Chen J. (2018). Study on the Role and Mechanism of Nrf2-ARE Pathway in Leukoplakia Syndrome Virus Infection. Master’s Thesis.

[46] Surh Y.J., Kundu J.K., Na H.K. (2008). Nrf2 as a Master Redox Switch in Turning on the Cellular Signaling Involved in the Induction of Cytoprotective Genes by Some Chemopreventive Phytochemicals. Planta Medica.

[47] Nawaz M.A., Fukai S., Bhandari B. (2016). Effect of alkali treatment on the milled grain surface protein and physicochemical properties of two contrasting rice varieties. J. Cereal Sci..

[48] Wang T., Zhang H., Wang L., Wang R., Chen Z. (2015). Mechanistic insights into solubilization of rice protein isolates by freeze–milling combined with alkali pretreatment. Food Chem..

[49] Xia N., Wang J.M., Gong Q., Yang X.Q., Yin S.W., Qi J.R. (2012). Characterization and In Vitro digestibility of rice protein prepared by enzyme-assisted microfluidization: Comparison to alkaline extraction. J. Cereal Sci..

[50] Tan K., Zhang B., Ma H., Li S., Zheng H. (2019). Oxidative stress responses of golden and brown noble scallops Chlamys nobilis to acute cold stress. Fish Shellfish. Immunol..

[51] Chen Y.Y., Li C.L., Huang X.H. (2015). Effects of microcystin on activities of immune enzymes in the white shrimp *Litopenaeus vannamei*. J. Guangdong Ocean. Univ..

[52] Liang D., Wang J., Ray G.W., Yang Q., Tan B., Dong X., Chi S., Liu H., Zhang S. (2020). Effects of different dietary levels of soybean protein hydrolysates on the growth performance, antioxidant capacity and relative mRNA expression levels of juvenile hybrid grouper (*Epinephelus fuscoguttatus* ♀ × *Epinephelus lanceolatus* ♂). Aquac. Nutr..

[53] Takahashi M., Moriguchi S., Yoshikawa M., Sasaki R. (1994). Isolation and characterization of oryzatensin: A novel bioactive peptide with ileum-contracting and immunomodulating activities derived from rice albumin. Biochem. Mol. Biol. Int..

[54] Shu Y., Hong P., Tang D., Qing H., Donde O.O., Wang H., Xiao B., Wu H. (2019). Comparison of intestinal microbes in female and male Chinese concave-eared frogs (*Odorrana tormota*) and effect of nematode infection on gut bacterial communities. Microbiol. Open.

[55] Fečkaninová A., Koščová J., Mudroňová D., Schusterová P., Cingeľová Maruščáková I., Popelka P. (2019). Characterization of two novel lactic acid bacteria isolated from the intestine of rainbow trout (*Oncorhynchus mykiss*, Walbaum) in Slovakia. Aquaculture.

[56] He S.Q., Li R.M., Yang Q.H., Tan B.P., Dong X.H., Chi S.Y., Zhang S., Liu H.Y. (2021). Effects of dietary zinc on growth, serum non-specific immune indexes, disease resistance and intestinal flora structure in juvenile *Litopenaeus vannamei*. J. Fish. China.

[57] He Y.F., Chi S.Y., Tan B.P., Zhang H.L., Dong X.H., Yang Q.H., Liu H.Y., Zhang S. (2017). Effect of yeast culture on intestinal microbiota of *Litopenaeus vannamei*. J. Guangdong Ocean. Univ..

[58] Hooper L.V., Wong M.H., Thelin A., Hansson L., Falk P.G., Gordon J.I. (2001). Molecular Analysis of Commensal Host-Microbial Relationships in the Intestine. Science.

[59] Yu W.N., Dai W.F., Tao Z., Xiong J.B. (2018). Characterizing the compositional and functional structures of intestinal microflora between healthy and diseased *Litopenaeus vannamei*. J. Fish. China.

[60] Zhang X., Li M., Tao X., Yang Y., Sun P., Jin M., Zhou Q., Jiao L. (2022). Effects of dietary montmorillonite supplementation on the growth performance, antioxidant capacity, intestinal barrier and microbiota composition in *Marsupenaeus japonicus*. Aquaculture.

[61] Li F., Xiang J. (2013). Signaling pathways regulating innate immune responses in shrimp. Fish Shellfish. Immunol..

[62] Gustilatov M., Widanarni, Ekasari J., Pande G.S.J. (2022). Protective effects of the biofloc system in Pacific white shrimp (*Penaeus vannamei*) culture against pathogenic Vibrio parahaemolyticus infection. Fish Shellfish. Immunol..

